# Advanced Surface Modifications for Blood-Contacting Surfaces of Medical Devices

**DOI:** 10.1155/2012/291735

**Published:** 2012-07-05

**Authors:** Zhongjun J. Wu, Narayana Garimella, Rolf Larsson, Eduard Brynda

**Affiliations:** ^1^Artificial Organs Laboratory, Department of Surgery, University of Maryland, Baltimore, MD 21201, USA; ^2^Department of Immunology, Genetics and Pathology, Uppsala University, 75185 Uppsala, Sweden; ^3^Department of Polymer Membranes, Institute of Macromolecular Chemistry, Academy of Sciences of the Czech Republic, 16206 Prague, Czech Republic

Surface modification of biomaterial interfaces remains an active field of research with a clear aim to enhance and retain blood compatibility of blood-contacting medical devices. Issues such as activation of coagulation pathways, altered hemostasis, inflammatory responses, and thrombosis are potential clinical complications during blood interaction with artificial surfaces that needs to be addressed when a new device is developed or there is a modification of a device or implant in the form of material, surface characteristics, shape, or function.

Large surface areas in certain devices, such as blood oxygenator membranes, hemodialysis membranes, or nanosized particles, present unique surface-related challenges. Minimizing pro-coagulant protein adsorption, platelet activation and deposition on the biomaterial interface are key factors that ultimately decide the performance and long-term reliability of a device. Among various types of biomedical devices, polymeric ones have a lion's share and accordingly substantial attention is being paid to surface-modification of polymeric surfaces. Several challenges in attaining modification of the surfaces include, but are not limited to, device specifics, application specifics and underlying thermodynamic and kinetic specific parameters.

International Journal of Biomaterials has launched this special issue with emphasis on analyzing numerous strategies in light of the ongoing advancements in design and development of blood-contacting medical devices. Peer-reviewed, original research, and review papers in the area of blood-compatible surface advancements are included. The topics comprise new methods and combination of principles, novel evaluation techniques, and current and future directions. The papers in this issue address several aspects of blood-contacting surfaces including modulation of interfacial bioactivities, evaluation methods and improvements in surface modification techniques.

Altogether, five research papers and one review paper of this special issue are concerned with materials, devices, parameters, and mechanisms in relation to blood compatibility. P. A. Patston et al. describe modulation of specific blood contact activation parameter (kallikrein) by C1-inhibitor in the presence of type IV collagen. They not only demonstrate the tight binding of C1-inhibitor with type IV collagen but also show collagen's influence in reducing the rate of inhibition. This paper provides an additional insight into collagen containing biomaterial surfaces. B. Dhandayuthapani and coworkers report on critical evaluation of nanocomposite scaffolds comprising single wall carbon nanotubes and Zein fibers. This paper covers structural, physicochemical, and hemocompatibility assessments that bring into focus the applicability of these unique composite scaffolds. M. Faria et al. contribute with another detailed evaluation in particular of surfaces composed of poly(ester urethane urea) oxygenation membranes. This paper incorporates cross-sectional studies of membranes and analyses to assess physicochemical and hemocompatibility features of their surfaces. Oligonucleotide and Parylene surface coating for a specific hemocompatible surface modification is described by M. Schleicher et al. in a separate paper. This is an interesting contribution in the direction of biofunctionalization of vascular implant surfaces. W. Van Oeveren and coworkers present a comparative study between different blood circulation models. They estimate functional as well as mechanical blood damage features under specific conditions. The review article of this special issue focuses on physicochemical and biological methods of surface modifications related to surfaces of cardiovascular implants. It is contributed by A. de Mel et al. The paper highlights the role of nanotechnology in connection with surface modification.

We are confident that this special issue provides a comprehensive review as well as new insights related to surface modification and evaluation technologies for blood contacting applications.

*Zhongjun J. Wu* *Zhongjun J. Wu* *Narayana Garimella* *Narayana Garimella* *Rolf Larsson* *Rolf Larsson* *Eduard Brynda* *Eduard Brynda*

